# Toward a mechanistic understanding of vulnerability to hook‐and‐line fishing: Boldness as the basic target of angling‐induced selection

**DOI:** 10.1111/eva.12504

**Published:** 2017-08-19

**Authors:** Thomas Klefoth, Christian Skov, Anna Kuparinen, Robert Arlinghaus

**Affiliations:** ^1^ Department of Biology and Ecology of Fishes Leibniz‐Institute of Freshwater Ecology and Inland Fisheries Berlin Germany; ^2^ Angling Association of Lower Saxony (Anglerverband Niedersachsen e.V.) Hannover Germany; ^3^ National Institute of Aquatic Resources (DTU Aqua) Technical University of Denmark Silkeborg Denmark; ^4^ Department of Biological and Environmental Science University of Jyväskylä Jyväskylä Finland; ^5^ Faculty of Life Sciences Department for Crop and Animal Sciences Division of Integrative Fisheries Management Humboldt‐Universität zu Berlin Berlin Germany

**Keywords:** angling, catchability, evolutionary change, growth, selection

## Abstract

In passively operated fishing gear, boldness‐related behaviors should fundamentally affect the vulnerability of individual fish and thus be under fisheries selection. To test this hypothesis, we used juvenile common‐garden reared carp (*Cyprinus carpio*) within a narrow size range to investigate the mechanistic basis of behavioral selection caused by angling. We focused on one key personality trait (i.e., boldness), measured in groups within ponds, two morphological traits (body shape and head shape), and one life‐history trait (juvenile growth capacity) and studied mean standardized selection gradients caused by angling. Carp behavior was highly repeatable within ponds. In the short term, over seven days of fishing, total length, not boldness, was the main predictor of angling vulnerability. However, after 20 days of fishing, boldness turned out to be the main trait under selection, followed by juvenile growth rate, while morphological traits were only weakly related to angling vulnerability. In addition, we found juvenile growth rate to be moderately correlated with boldness. Hence, direct selection on boldness will also induce indirect selection on juvenile growth and vice versa, but given that the two traits are not perfectly correlated, independent evolution of both traits is also possible. Our study is among the first to mechanistically reveal that energy‐acquisition‐related behaviors, and not growth rate per se, are key factors determining the probability of capture, and hence, behavioral traits appear to be the prime targets of angling selection. We predict an evolutionary response toward increased shyness in intensively angling‐exploited fish stocks, possibly causing the emergence of a timidity syndrome.

## 1 INTRODUCTION

A growing body of literature has drawn attention to the potential for intensive and/or size‐selective commercial fisheries to act as an evolutionary force altering a range of life history traits, such as reproductive investment, size and age at maturation, and genetic growth capacity (reviewed in Jørgensen et al., 2007; Laugen et al., 2014; Heino, Díaz Pauli, & Dieckmann, 2015; Kuparinen & Festa‐Bianchet, 2017). Recent studies have also addressed the question of fisheries‐induced adaptive changes in the context of recreational fishing, largely confirming the findings from commercial fisheries studies. Accordingly, intensive and/or size‐selective recreational fishing leads to increased reproductive investment and reduced age and size at maturation, which collectively reduces adult size at age (Alós, Palmer, Catalan, et al., 2014; Arlinghaus, Matsumura, & Dieckmann, 2009; Matsumura, Arlinghaus, & Dieckmann, 2011; Saura et al., 2010). Moreover, work in largemouth bass (*Micropterus salmoides*) selected for high and low vulnerability to angling has revealed genetically based changes in behavioral traits such as aggression and vigilance during parental care (Philipp et al., 2009; Sutter et al., 2012), but clear documentation of evolution of behavioral traits as a consequence of high angling pressure is still missing (Arlinghaus et al., 2017; Diaz Pauli & Sih, 2017; Heino et al., 2015).

Passive fishing gear should directly select on behavioral traits related to exploration, activity, boldness, or aggression because these traits directly affect exposure of individual fish to the fishing gear by increasing encounters or promote the ingestion probability of baits or lures (Alós, Palmer, & Arlinghaus, 2012; Arlinghaus et al., 2016, 2017; Biro & Post, 2008; Diaz Pauli & Sih, 2017; Enberg et al., 2012; Lennox et al., 2017; Uusi‐Heikkilä, Wolter, Klefoth, & Arlinghaus, 2008). Direct selection on behavioral traits can also indirectly change growth rate and other life history traits as long as these traits are heritable and correlated with the behavioral trait under selection (Biro & Post, 2008; Biro & Sampson, 2015; Uusi‐Heikkilä et al., 2008). Although strong selection pressures acting on behavioral traits in recreational fisheries are supported by theoretical arguments and simulation models (Alós et al., 2012; Andersen, Marty, & Arlinghaus, 2017; Enberg et al., 2012; Uusi‐Heikkilä et al., 2008), few experimental studies on this topic exist so far. The majority of these support the assumption of positive correlations between exploration, habitat choice behavior, activity, aggression, boldness and intensity of parental care, and vulnerability to hook‐and‐line fisheries (Alós, Palmer, Rosselló, & Arlinghaus, 2016; Alós, Palmer, Trias, Díaz‐Gil, & Arlinghaus, 2015; Härkönen, Hyvärinen, Niemelä, & Vainikka, 2015; Härkönen, Hyvärinen, Paappanen, & Vainikka, 2014; Klefoth, Skov, Krause, & Arlinghaus, 2012; Monk & Arlinghaus, 2017a; Sutter et al., 2012; Wilson, Brownscombe, Sullivan, Jain‐Schlaepfer, & Cooke, 2015). Following the timidity syndrome hypothesis recently put forward by Arlinghaus et al. (2016, 2017), we expected to find a particularly clear relationship of risk‐taking behavior (i.e., boldness) and vulnerability to hook‐and‐line fisheries.

Most fishing gears are positively size‐selective for physical (gape size) and managerial reasons (size‐based harvest limits) (Lewin, Arlinghaus, & Mehner, 2006). Moreover, larger fish of some species can be more vulnerable to hook‐and‐line or other passive gear types due to underlying behaviors, for example, increased dominance or elevated activity and space use that increase encounters with the gear or readiness to take a baited hook (Biro & Post, 2008; Tsuboi, Morita, Klefoth, Endou, & Arlinghaus, 2016). Size‐selective harvesting is so common in most fisheries that it has prompted the “intuition” (Walters & Martell, 2004) among many that fisheries‐induced evolution of slow growth should generally be expected (see Enberg et al., 2012 for alternative views). Supporting this argument, the heritability of growth rate is at least moderate in fishes (Garcia de Leaniz et al., 2007; Gjedrem, 1983), and therefore, selective harvesting of the fast growing portion of a fish population over several generations can lead to evolutionary downsizing (Alós, Palmer, Catalan, et al., 2014; Conover & Munch, 2002; Matsumura et al., 2011; Swain, Sinclair, & Hanson, 2007; Uusi‐Heikkilä, Sävilammi, Leder, Arlinghaus, & Primmer, 2017; Uusi‐Heikkilä et al., 2015) as long as the selection pressures induced by fishing on size or correlates of body size (e.g., age and size at maturation) are larger than natural selection pressures acting in potentially opposite directions (Carlson et al., 2007; Edeline et al., 2007; Enberg et al., 2012). However, any observed changes in adult growth rate can also be a consequence of altered maturation schedules or increased reproductive investment, without necessarily involving changes in the general growth capacity of the organism (Alós, Palmer, Catalan, et al., 2014; Enberg et al., 2012; Heino et al., 2008; Uusi‐Heikkilä et al., 2015). Obviously, changes in adult growth rate may also be caused by fisheries‐induced evolution of juvenile growth rate. Because no energy is channelized into gonad tissue in juveniles, their growth rate constitutes a clean measure of growth capacity in fishes, and it is possible that juvenile growth rate either decreases or increases in response to fishing mortality depending on the intensity of selection (i.e., mortality), the degree of size selection, and the opportunity to reap fitness benefits late in life (Enberg et al., 2012; Matsumura et al., 2011). Using experimentally fished crayfish (*Cherax destructor*), Biro and Sampson (2015) showed that trapping selectively captured fast growing juvenile crayfish and that fast growth was strongly correlated with boldness. Hence, evolution of juvenile growth may be directly caused by selection acting on behavior, which in turn might alter postmaturation growth independent of any changes in maturation schedules. To better understand the direction of evolutionary changes to be expected from fishing, an understanding of the mechanistic basis of fishing selection and whether selection operates mostly on life history or on other traits (such as behavioral traits) is needed (Uusi‐Heikkilä et al., 2008; Lennox et al., 2017).

In addition to behavior and potentially life history, morphological variables can also affect the likelihood of capture and therefore contribute to the selective properties of recreational fishing. Beyond the obvious size‐selectivity mentioned before, Alós, Palmer, Linde‐Medina, and Arlinghaus (2014) found that more streamlined coastal fish and fish with larger mouth gape were more likely to be captured than deeper bodied fish and fish with small mouth gaps. These findings could represent correlations of body shape and swimming activity (Haas, Heins, & Blum, 2015) or relate to physical aspects of foraging in relation to hook size and gape‐size limitations. Therefore, following arguments by Uusi‐Heikkilä et al. (2008) and Lennox et al. (2017), we expected that behavioral, life history, and morphological traits should jointly determine the vulnerability of individual fish to passively operating angling gear.

We used juvenile carp (*Cyprinus carpio*) of identical age and a narrow size range as a model species to test for the strength and direction of selection acting on boldness‐related behaviors, growth, and morphological characteristics in a passively operated angling fishery. Our objectives were to shed light on the behavior‐based mechanisms underlying vulnerability to angling and to disentangle the relative importance of behavior and (juvenile) growth for affecting vulnerability to angling. We hypothesized that resource‐acquisition‐related behaviors constitute key traits under selection in passively operating angling fisheries for carp (Arlinghaus et al., 2017). Accounting for boldness should thereby capture a relevant portion of direct selection acting on body size or growth rate.

## 2 MATERIAL AND METHODS

We performed a pond experiment designed to quantify the capture probability‐related selection gradients on key behavioral and morphological traits as well as juvenile growth rate in recreational angling using juvenile carp (*Cyprinus carpio*) as a model species. To derive consistent behavioral traits that characterize the personality (e.g., boldness) of *N* = 120 individual carp, a range of behavioral traits, such as activity in ponds and the use of feeding arenas, were assessed after release in three replicated semi‐natural ponds in a group context. Just before release, standardized pictures of the fish were taken for analyses of geometric morphometrics. The vulnerability of the test fish to passive angling tactics was tested in angling trials lasting 7 days and 20 days, and at the end of the experiment, we measured the expressed growth rate of the experimental fish in the ponds as a measure of juvenile growth capacity assuming that the feed we delivered was ad libitum.

### 2.1 Experimental fish

All carp were raised in a commercial fish hatchery (Fischzucht Wegert, Ostercappeln, Germany, 52°19′52′′N, 8°14′48′′E) in the same common‐garden pond environment. About 40 phenotypically scaled parental carp were stocked into a monoculture pond in spring. Spawning and breeding occurred naturally. The emerging young‐of‐the‐year carp consisted of scaled and mirror carp phenotypes, which were fed with standard carp dry food (1–3 mm diameter, Aller Classic, Aller Aqua, Golßen, Germany) in addition to natural food developing in the shallow (1.5 m deep) earthen breeding pond (40 m × 50 m). The pond was fed with water from a nearby creek (Caldenhofer Graben). When the fish reached an age of about 10 months, the pond was drained, and a random sample of scaled and mirror carp phenotypes was transported to the Leibniz‐Institute of Freshwater Ecology and Inland Fisheries in Berlin, Germany. There, fish were initially kept in indoor tanks (1 m × 1 m × 1 m, 5 fish per 1,00l) fed with tap water (mean temperature ± *SD* 18 ± 1.5°C, exchange rate once per day) for 5 weeks until experiments started. During this holding period, about 1% of the fish died. Fish were exclusively fed with standard carp pellets (5 mm diameter, Aller Classic, Aller Aqua, Golßen, Germany) at a maintenance ratio of about ~1.5% of fish body wet mass per day. Before experiments started, fish were slowly acclimatized to water temperatures within the test environments (ponds) by altering the temperature at a maximum of 1°C per day (Pitt, Garside, & Hepburn, 1956). The maximal total change in temperature the fish experienced over the acclimatization period was 3°C.

### 2.2 Assessment of personality, morphology, and vulnerability to angling in ponds

**Figure 1:**
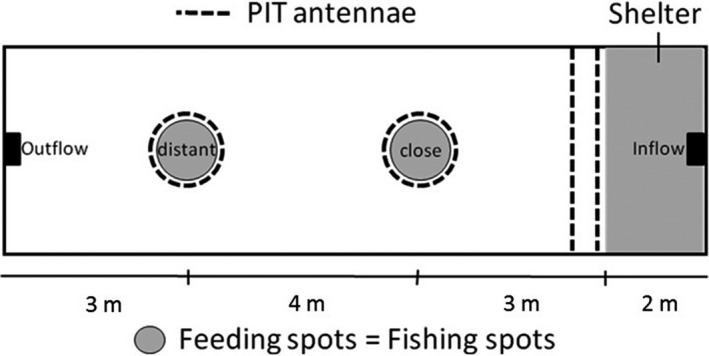
Experimental setup for behavioral observations under semi‐natural pond conditions. Within the ponds, boldness was defined in a group setting by low sheltering times and high number of visits at the close and the distant feeding spot (circles). All structures within the ponds were covered by passive integrated transponder antennae (PIT)

Behavioral experiments were designed to assess the boldness‐related personality of angling‐naïve carp in a semi‐natural pond environment in groups, which in contrast to laboratory experiments has previously been found to yield reliable personality data in carp (Klefoth, 2017; Klefoth et al., 2012). Before release, we surgically implanted passive integrated transponder (PIT) tags (23 mm length, 2 mm width, Oregon RFID, Oregon, USA) into the fish's body cavity following the procedure outlined in Skov et al. (2005). All ponds were equipped with several PIT tag antenna loops (Oregon RFID, Oregon, USA) that were able to detect the PIT tags (Figure 1; Appendix S2). During PIT tag surgery, fish were anaesthetized using 1 ml/L of 9:1 solution of ethanol:clove oil in well‐aerated water at 18°C. After surgery, fish were measured for total length (TL, to the nearest 1 mm), weight to the nearest g, and standardized pictures were taken from both sides of the fish's body for geometric morphometrics analyses (Nikon DX40 mounted approximately 45 cm above the fish on a fix stand). Before pictures were taken, fish were placed in a straight position and the fins were stretched.

Stationary passive telemetry systems within three replicated experimental ponds (12 m × 5 m × 1 m, L × W × H, Figure 1) were simultaneously used to enumerate carp behavior in groups. Each of three ponds was stocked with *N* = 40 randomly selected carp (total *N* = 120, mean TL ± *SD* pond 1: 199 ± 9.7 mm, pond 2: 199 ± 9.0 mm, pond 3: 198 ± 9.0 mm). Carp were held in monoculture with no fish predators, but ponds were regularly visited by fish‐eating birds. The ponds were continuously supplied with unfiltered lake water (about 1 L/s) from the nearby Müggelsee in Berlin (52°26′57′′N, 13°38′59′′E), which is a large (800 ha) natural lake. The bottom of the ponds consisted of a mixture of gravel (5–20 mm), sand, and mud. Within this substrate, benthic invertebrates that were flushed into the ponds with the supply water were regularly observed. Thus, the ponds were assumed to constitute a semi‐natural environment. The bank of the ponds consisted of perforated bricks, and the bank inclination was about 45°. A shelter structure (rectangular area of the pond [2 m × 5 m]) made out of black plastic, and two open feeding spots (0.5 m diameter) in different distances to the shelter were installed (see Figure 1 and Klefoth et al., 2012 for the full description of the experimental setup). The feeding spots were later also used as angling sites. As argued in Klefoth et al. (2012), the shelter structure was assumed to be the safest habitat within each pond as it provided refuge and no possibility for bird predation events. To reach the feeding spots, the fish had to cross a comparably large open area, similar to a standard open‐field test used to measure boldness in laboratory environments with fishes (Budaev, 1997). Both shelter and feeding spots were covered by PIT antennae (Figure 1) enabling the quantification of the individual number of visits at the two feeding spots and the time spent sheltering as three measures of boldness (Klefoth et al., 2012). Low scores of the refuge time and large numbers of visits on feedings spots were assumed to indicate boldness. Functionality of the PIT system was confirmed prior to pond experiments (Appendix S2). Fish were allowed to acclimatize for 2 days before a behavioral observation period of 6 days started. During the six‐day initial personality assessment period, fish were fed daily (5 mm carp pellets, Aller Classic, Aller Aqua, Golßen, Germany) with a total amount of 1% of the pond's population mass (assessed at the release time). Feeding took place from 2 hr before sunset until 2 hr after sunset on an hourly basis while alternating between the two feeding spots to control for potential impacts of daytime and site on boldness measurements.

To assess the angling vulnerability of individual carp carrying specific phenotypes, experimental carp angling was conducted for seven consecutive days (short‐term vulnerability) followed by angling for another 13 consecutive days (20 days in total, referred to here as long‐term vulnerability) after the initial six‐day observation period. Carp were angled every day for four consecutive hours, and the angling location alternated between the close and the distant feeding spot on an hourly basis. The complete procedure followed the angling protocol described by Klefoth, Pieterek, and Arlinghaus (2013). The baited hooks were not placed randomly within the pond environment to standardize the fishing procedure and to ensure comparability to former studies (Klefoth et al., 2012; Klefoth et al., 2013). Moreover, in actual carp fishing the use of prebaited fishing spots is common (Arlinghaus & Mehner, 2003), hereby our method resembled what would be expected under real angling conditions. Further, benthic invertebrates were available as alternative food for the fish, thus individual carp were able to choose to forage on either artificial and/or natural food items. To further assure comparable ability of all fishes to access the baits, angling locations were regularly alternated between the close and the distant feeding spot. Sweet corn was used as bait offered on a standard bolt‐rig, which is known to result in 100% of shallow hooking in the mouth region (Rapp, Cooke, & Arlinghaus, 2008). Materials used for angling followed standard practice in specialized carp angling (Arlinghaus & Mehner, 2003) but scaled to small carp (3‐kg monofilament line, 15 g sinker, fishing rod with 0.3 lb test curve). Landed fish were identified by the PIT tag (Pocket reader, Allflex, Dallas, Texas, USA). Afterward, fish were immediately released back into the pond. This procedure lasted a maximum of 30 s.

After the 20‐day angling period ended, we continued to feed the fish with 1% of their initial population body mass per day for another 30 days to determine growth of the juvenile fish. The feeding procedure followed the same protocol as conducted during the undisturbed behavioral observations prior to angling. Then, the ponds were drained and fish were again measured for their total length to assess growth increment.

The mean water temperature ± *SD* in the ponds during undisturbed behavioral observations and the first 7 days of angling was 19.0 ± 0.5°C (range: 17.0–20.2°C). Mean water temperature ± *SD* during angling days 8–20 dropped and was 14.9 ± 0.9°C (range: 13.9–17.0°C). The temperature was 13.3 ± 1.3°C (range: 11.2–16.2°C) during the subsequent feeding period without angling.

After draining the ponds, *N* = 94 carp provided a full dataset starting with PIT implantation until completed growth measurements (78.3% of the initial stock). The other 26 individuals disappeared due to (most likely bird or otter) predation (*N* = 11, 9.2%) or lost their PIT tags (*N* = 15, 12.5%), which is known to be a problem in carp tagging studies (Økland, Hay, Næsje, Nickandor, & Thorstad, 2003). As indicated by our PIT system data, mortalities and tag loss mainly occurred during the last 2 weeks of the additional feeding period (when predators were less disturbed by angling activities), and mortalities were similarly distributed between the ponds (either three or four individuals died in each pond). Therefore, food distribution among individuals remained constant over the complete experimental period.

### 2.3 Statistical analyses

#### 2.3.1 Pond behavior

Using the raw PIT detection data, three boldness‐related measures characterizing individual carp were derived following the protocols described in Klefoth et al. (2012). For each individual fish, the mean “time spent sheltering” per day (expressed as mean minutes/hr) and the mean “number of visits at the feeding spots” per day (expressed as mean #/hr) were estimated, the latter separately for the close and the distant feeding spot. The repeatability of behaviors within ponds was estimated using Spearman correlations and additionally following Lessells and Boag (1987) using mean values from the first week (behavioral observation without angling) and the second week (7 days of angling), separately. For subsequent analyses of angling‐induced selection on behavior, mean values for each of the three boldness measures per individual fish during the first week of pond behavior undisrupted by angling were estimated. A correlation matrix for all variables included in the analyses and comprising the correlation of boldness prior to the onset of angling and growth as determined over 58 days in ponds was calculated using Pearson's correlations.

#### 2.3.2 Morphological traits

The body shape and the shape of the head of each individual were examined as morphological traits potentially correlated with angling vulnerability using a landmark‐based assessment approach (Rohlf & Marcus, 1993). To that end, we digitized a total of 16 landmarks on the left side of each specimen using the tpsDig2 software (http://life.bio.sunysb.edu/morph) (Appendix S1). The landmarks were as follows: (i) tip of the upper jaw, (ii) posterior corner of the upper jaw, (iii) corner of the insertion of the pectoral fin, (iv) insertion of the pelvic fin, (v) anterior insertion of the anal fin, (vi) posterior insertion of the anal fin, (vii) ventral point of maximum curvature of the peduncle, (viii) posterior extremity of the lateral line, (ix) dorsal point of maximum curvature of the peduncle, (x) posterior insertion of the dorsal fin, (xi) anterior insertion of the dorsal fin, (xii) dorsal insertion of the head, (xiii) dorsal edge of head perpendicular, (xiv) center of the eye, (xv) ventral edge of head perpendicular, and (xvi) posterior end of operculum (Appendix S1). Raw co‐ordinates were superimposed using general Procrustes superimposition in software MorphoJ 1.03 (Klingenberg, 2011). To eliminate potential effects of dorsoventral bending (called arching), Burnaby's orthogonal projection following Valentin, Penin, Chanut, Sévigny, and Rohlf (2008) was applied. The explained variances of the subsequent PCA analyses were reduced by less than 5% as a consequence of the correction procedure, indicating low bending of the photographed fish. Arching‐free shape descriptors were then used for subsequent analyses. Principal component analyses (PCA) of Procrustes shape co‐ordinates were performed separately using MorphoJ. To further investigate potential impacts of the head morphology on angling vulnerability (Alós, Palmer, and Linde‐Medina, 2014), landmarks 1, 2, 12, 13, 15, and 16 were separately analyzed (Appendix S1). We used data from the resulting first principal components, which explained 13.4% (full body shape) and 43.6% (head shape) of the variation. To control for the effect of size on morphology, residuals of linear regressions between factor scores of the first principal components and total length were calculated and used for further selection analyses.

#### 2.3.3 Juvenile growth rate

All fish were raised in the same common garden under natural conditions and were descendants of the same pool of parental fish. Afterward, all fish experienced the same holdings conditions and the same food levels. Because environmental conditions were equal for all fish prior to experimentation, differences in size between individuals at the onset of the experiment already reflected differences in growth over the life span. Thus, size of the fish (TL, mm) was interpreted as a surrogate for growth and used as a predictor variable to calculate fitness in the angling fishery. Further, absolute growth increments (mm) over a 58 day period were calculated. Because fishing may select on growth via behavior (Biro & Sampson, 2015), potentially correlated effects of boldness on growth were separated using residuals of a linear regression between growth increment and boldness in ponds (visits at the distant feeding spot) for further analyses.

#### 2.3.4 Mean standardized selection gradients (β_μ_) induced by angling on adaptive traits

In a fishing context, the survival component of fitness is defined by the capture event, which usually ends in death by harvest. Accordingly, a fish was considered theoretically dead (coded as fitness of zero) if it was captured in the experimental fishing, and otherwise considered alive (coded one). Individual recaptures that occurred during experimental angling were not considered further. We used a nested logistic regression approach considering individual fish nested within replicated ponds to analyze predictors of survival of carp exposed to an angling fishery using boldness‐related behaviors, morphology, and growth (TL and length increment over 58 days) as predictors. All predictor variables were z‐standardized to a mean of 0 and a *SD* of 1 prior to inclusion into the regression model. A total of six predictor variables were analyzed to determine survival as a measure of fitness of the carp. These variables were as follows: (i) total length at the time of stocking within ponds (*TL*); (ii) body shape (*SB*) and (iii) head shape (*SH*), both based on the morphological analyses; (iv) number of visits at the close and the distant feeding spot within ponds as an indicator of boldness under semi‐natural conditions in groups (*BP*); (v) time spent sheltering within ponds as a further measure of boldness in ponds (*SP*); and (vi) growth rate in ponds (residuals) over 58 days (*G*). In case of the “*BP*” variable, only the distant feeding spot was ultimately considered in the final models. This was done because the number of visits at the close and the distant feeding spot were highly correlated (Pearson's correlation between the close and the distant feeding spot *r* = .887, *p* < .001), and the distant feeding spot was assumed to have been perceived as particularly risky by the fish as shown in previous experiments (Klefoth et al., 2012). Our starting model was:

logit(s)^ ^= α_0_ + α_1_ × *BP* + α_2_ × *TL* + α_3_ × *G *+ α_4_ × *S *+ α_5_ × *SH* + α_6_ × *SP* + α_7_ × *G²* + α_8_ × *BP²*.

Two different models with the same independent variables were calculated, as fitness (i.e., survival of an angling fishery) was based on either “short” (7 days) or “long” (20 days) angling durations. All models for both datasets also contained quadratic terms for boldness in ponds and for two measures of growth (“TL” and “G”) to test for stabilizing or disruptive selection on these traits (Olsen & Moland, 2011). The most parsimonious models were selected based on Akaike's information criterion corrected for small sample sizes AIC_c_ (Burnham, Anderson, & Huyvaert, 2011) and based on AIC_c_ weights _*wi*_(AIC_c_) calculated following the instructions by Wagenmakers and Farrell (2004). We compared the AIC_c_ scores and weights between a restricted set of models based on their relevance to explain carp survival fitness in our experiment rather than testing all possible combinations of predictor variables (Burnham & Anderson, 1998; see also Olsen, Heupel, Simpfendorfer, & Moland, 2012 for a similar approach in a comparable field study). For the best models, the total amount of explained variances was calculated using Nagelkerke's pseudo *R*².

Multivariate regression models on relative fitness or fitness components such as survival allow the interpretation of regression coefficients as selection gradients following the landmark work by Arnold and Wade (1984). We estimated mean standardized selection gradients (β_μ_) based on (linearized, Janzen & Stern, 1998) logistic regression coefficients to allow comparisons of selection strengths caused by angling among traits carrying different units following the methods described in Matsumura, Arlinghaus, and Dieckmann (2012). To that end, logistic regression coefficients for all adaptive traits from the final models were transformed to their linear equivalents following Janzen and Stern (1998). The resulting unstandardized selection gradients represented the *SD*‐standardized selection gradients because traits were initially standardized to a mean of zero and a *SD* of 1 (Matsumura et al., 2012). To estimate β_μ_ as unitless measures of strength of selection, selection gradients were multiplied by the original mean and divided by the original *SD* of the phenotypic trait (Matsumura et al., 2012). The β_μ_ is preferred for representing selection in the wild, and it represents the relative change in fitness that results from doubling of the trait value (Matsumura et al., 2012). The measure allows comparisons of the strength of selection acting on several traits that differ in units, means, and variance (Hereford, Hansen, & Houle, 2004; Matsumura et al., 2012).

Logistic regression analyses were conducted using the software package R version 3.1.2 (R Development Core Team) by applying the library lme4 (Bates, Maechler, Bolker, & Walker, 2014), and AIC_c_ values were calculated using library AICcmodavg (Mazerolle, 2013). Pearson's and Spearman rank correlations applied were conducted using software package SPSS 20.

## 3 RESULTS

### 3.1 Personality of individual carp assessed in groups in ponds

Boldness‐related carp behavior in the ponds assessed in groups was not or only moderately correlated with all other variables (Table 1) and was found to be highly consistent and repeatable, indicating personality with respect to boldness (Table 2). Repeatability estimates for all boldness measures (visits of feeding spots and use of the shelter) were high and significant, ranging between *r* = .53 and *r* = .74, with significant underlying *F*‐statistics and Spearman correlations in all cases (Table 2).

**Table 1:** Correlation matrix of z‐standardized variables involved in the pond experiment

Trait	BP	TL	G	SB	SH	SP
BP	1	0.100	0.310	−0.248	−0.148	−0.521
TL		1	0.047	0	0	−0.024
G			1	−0.129	−0.133	−0.191
SB				1	−0.164	0.090
SH					1	0.037
SP						1

BP, number of visits at the distant feeding spot within ponds, TL, total length at the time of stocking within ponds, G, growth rate in ponds over 58 days, SB, body shape, SH, head shape, SP, time spent sheltering within ponds.

**Table 2:** Rank‐order consistency and repeatability of boldness‐related measures of carp within the pond environment (*N* = 94)

Rank‐order consistency				Repeatability		
Variable	*N*	Spearman *r*	*p*	*F*	*p*	*r*
Close feeding spot	94	.789	<.001	2.322	<.001	.58
Distant feeding spot	94	.746	<.001	2.101	<.001	.53
Shelter use	94	.647	<.001	3.673	<.001	.74

### 3.2 Angling vulnerability

During the first 7 days of angling, 38 of 94 individuals were captured (40% of the total population, 40.1 ± 6.3% per pond, *N* = 3) within 84 rod‐angling hours. Over 20 angling days at 240 rod‐angling hours, a total of 49 carp was captured (53% of the total population, 51.8 ± 6.1% per pond, *N* = 3). Catch per unit effort (CPUE based on rod‐angling hours) was 0.46 fish/hr during the first 7 days (short‐term vulnerability), and 0.21 fish/hr over the complete course of the experiment (long‐term vulnerability).

**Figure 2:**
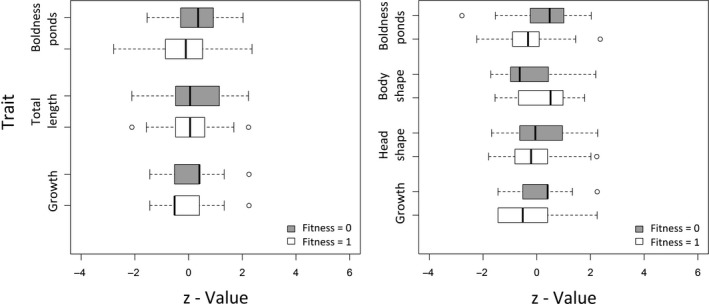
Box‐plots comparing z‐standardized trait values between vulnerable (fitness = 0, gray) and invulnerable (fitness = 1, white) carp identified in regression models to be under selection in a 7 days lasting passive angling fishery (left) and in a 20 days lasting passive angling fishery (right). Boxes define the 25th and 75th percentiles, and median values are indicated by dark black bars within the boxes

The captured individuals were on average larger, grew faster, and behaved more boldly compared to their uncaught conspecifics (Table 3). In the first 7 days of angling, the best model explaining survival‐based fitness of carp consisted of size (*TL*), growth (*G*), and boldness within ponds (*BP*) (Tables 4, 5; Figure 2). Analyzing 20 days of angling revealed three models within a narrow ∆AIC_c_ range of 0.8, which was similarly supported by AIC_c_ weights (Table 4). For example, AIC_c_ weights of the best fitting model (_*wi*_(AIC_c_) = 0.341) were 3.7 times higher (and therefore 3.7 times more likely to be the best model) compared to the fourth best model (_*wi*_(AIC_c_) = 0.092) (Table 4). These three best models included boldness (*BP*) and growth (*G*) in all cases, and body shape (*SB*) and size of the head and mouth (*SH*) (in two and one cases, respectively) to best explain fitness in the carp fishery (Tables 4, 5; Figure 2; Appendix S1). Note that in the long‐term fishery, the size of the fish (*TL*) was no longer present in the best‐supported models.

**Table 3:** Mean ± *SD* values of different behavioral data, total length, and growth for caught and uncaught individuals in a passive angling fishery from the pond experiment with 7 days and 20 days of angling

Trait	Captured Mean ± *SD*	Not Captured Mean ± *SD*
Short‐term angling (7 days)	*N* = 38	*N* = 56
Time spent sheltering (min/hr)	5.7 ± 2.2	6.7 ± 2.8
Number of visits at the close feeding spot (#/hr)	5.3 ± 1.1	4.3 ± 1.6
Number of visits at the distant feeding spot (#/hr)	5.0 ± 1.2	4.3 ± 1.6
Total length (mm)	201.6 ± 10.0	198.0 ± 8.4
Growth 58 days (mm)	9.3 ± 5.1	6.8 ± 5.4
Long‐term angling (20 days)	*N* = 49	*N* = 45
Time spent sheltering (min/hr)	6.0 ± 6.6	6.6 ± 2.5
Number of visits at the close feeding spot (#/hr)	5.3 ± 1.3	4.2 ± 1.6
Number of visits at the distant feeding spot (#/hr)	5.0 ± 1.4	4.0 ± 1.5
Total length (mm)	200.1 ± 10.7	198.8 ± 7.2
Growth 58 days (mm)	9.7 ± 5.3	5.8 ± 4.8

**Table 4:** Nested logistic regression of carp survival in ponds within 7 d and 20 d of angling showing the model structure, number of parameters (#P), AIC_c_ values, and AIC_c_ weights _*wi*_(AIC_c_)

Model no.	Model structure	#P	AIC_c_	_*wi*_(AIC_c_)
Short‐term angling (7 days)				
1	BP + TL + G + SB + SH + SP + BP² + G²	9	130.1	0.055
2	BP + TL + SB + SH + G + SP + G²	8	130.4	0.047
3	BP + TL + SB + SH + SP + G	7	129.6	0.071
4	BP + TL + SB + SH + G	6	128.0	0.157
5	BP + TL + SB + G	5	128.3	0.135
6	BP + TL + G	4	**126.3**	**0.368**
7	TL + G	3	129.2	0.086
8	G	2	130.5	0.045
9	NULL	1	131.0	0.035
Long‐term angling (20 days)				
1	BP + TL + SB + SH + G + SP + BP² + G²	9	130.0	0.004
2	BP + TL + SB + SH + G + SP + G²	8	127.4	0.015
3	BP + TL + SB + SH + G + SP	7	125.6	0.038
4	BP + TL + SB + SH + G	6	123.8	0.092
5	BP + SB + SH + G	5	**121.6**	**0.279**
6	BP + SB + G	4	**121.2**	**0.341**
7	BP + G	3	**122.0**	**0.228**
8	G	2	131.3	0.002
9	NULL	1	136.4	0.000

Bold values indicate models with the lowest AIC_c_, a ∆AIC_c_ < 1, and the greatest _*wi*_(AIC_c_). Explanatory variables include TL, total length at stocking; SB, body shape; SH, head shape; BP, number of visits at the distant feeding spot within ponds; SP, time spent sheltering within ponds; G, growth rate in ponds over 58 days.

**Table 5:** Angling‐induced selection acting on carp behavior, morphology, and growth in the pond experiment showing partial logistic regression coefficients (α), standard errors (*SE*), *p* values (*p*), mean standardized selection gradients (β_μ_), and pseudo R² values. The best models containing the most variables within a ∆AIC_c_ < 1 and the greatest _*wi*_(AIC_c_) in relation to the best models in bold in Table 4 are presented

Variable	α	*SE*	*p*	β_μ_	*R*²
Short‐term angling (7 days)					
Pond behavior (BP)	−0.518	0.24	.029	−0.437	.17
Total length (TL)	−0.373	0.23	.105	−3.422	
Growth (G)	−0.357	0.23	.117	−0.288	
Long‐term angling (20 days)					
Pond behavior (BP)	−0.768	0.24	.004	−0.655	.30
Body shape (S)	0.343	0.25	.169	−0.08 × 10^−6^	
Head shape (SH)	−0.340	0.25	.168	−9.77 × 10^−7^	
Growth (G)	−0.699	0.26	.007	−0.424	

Mean standardized selection gradients allowed direct comparisons of the strength and direction of angling‐induced selection acting on each of the traits included in the best‐supported survival models. During the short‐term angling fishery (7 days), the size of the carp (with large fish being more likely to be captured) was more than seven times more strongly under selection than boldness‐related behavioral traits within ponds, with bold fish being more likely to be captured than shy individuals (Tables 3, 5). Further, the morphological variable *TL* also exerted much greater influence on vulnerability than juvenile growth rate as measured in ponds (*G*) (Table 5). However, over the longer fishing period of 20 angling days, the largest normalized selection gradients were acting on the boldness of the fish (*BP*)—a value which was 1.5 times greater than the selection acting directly on growth (*G*). Here, *TL* of the fish no longer explained the survival of carp in the angling fishery (Table 4). Correlation analysis revealed the juvenile growth (*G*) of the fish to be moderately correlated with the number of visits at the distant feeding spot as a measure of pond boldness (*BP*) (Pearson's *r* = .310, *p* = .002; Table 1).

Overall, within our size‐restricted set of experimental fish, boldness‐related behavior was found to be the most important trait under selection by angling over a period of 20 days, whereas size (*TL*) and growth (*G*) had lower (as observed for *G*) or no (as observed for *TL*) importance for determining vulnerability to angling when fishing took place over a 20 days angling period (Tables 4, 5). Hence, angling selection acted directly and most strongly on resource acquisition‐related behavior and only secondarily on juvenile growth rate. Only negligible selection pressures were found to act on body shape (*SB*) and size of the head and mouth (*SH*) (Table 5; Appendix S1), and a lower fitness (i.e., higher vulnerability to angling) was revealed for more deeply bodied fish and for carp with larger heads. There was also no sign of disruptive selection as no quadratic terms were retained in the best‐supported models.

## 4 DISCUSSION

Our study provided strong support for the hypothesis that a passive fishery with hook and line directly selects on behavioral traits related to risk‐taking during foraging (i.e., boldness) as expressed by angling‐naïve groups of carp in semi‐natural replicated ponds. In fact, we found the standardized selection pressure on boldness to be much stronger than angling‐induced selection acting directly on juvenile growth rate so that one might expect a largely independent selection response to angling in boldness without a corresponding change in juvenile growth rate. In contrast to recent laboratory data presented on trapping‐induced selection on crayfish by Biro and Sampson (2015), we only found a modest correlation between boldness and growth. However, even this modest correlation might induce an indirect selection gradient on juvenile growth rate and might lead to a correlated selection response as previously argued by Biro and Post (2008) and Uusi‐Heikkilä et al. (2008). Our study joins other recent work emphasizing the importance of behavior in the context of fishing‐induced selection with passive gear types (e.g., Olsen et al., 2012; Alós et al., 2012, 2016; Alós, Palmer, Trias, et al., 2015; Tsuboi et al., 2016; Arlinghaus et al., 2017; Diaz Pauli & Sih, 2017; Monk & Arlinghaus, 2017a,b; Lennox et al., 2017) but is novel insofar as it reveals the relative importance of behavioral selection in comparison with other morphological and life history traits under semi‐natural conditions in free‐ranging fishes. Our work supports recent field studies who also revealed that total length was irrelevant in terms of contribution to individual variation in vulnerability to angling in a small‐bodied coastal fish species (Alós et al., 2016) and also in common carp under natural conditions (Monk & Arlinghaus, 2017b). Similar to our case, Alós et al. (2016) found selection to directly operate on home range and the intensity of exploring the home range, whereas Monk and Arlinghaus (2017b) did not detect any behavioral, morphological, or physiological predictors of individual vulnerability to angling of carp under natural conditions in a 25‐ha lake. The latter finding is noteworthy because Monk and Arlinghaus (2017b) also studied the intensity of food patch uses, but were not able to relate this behavior to vulnerability to capture. One possible reason is that the whole‐lake telemetry system used by Monk and Arlinghaus (2017b) is less spatially accurate as the PIT tag system used in the present work. Therefore, our measure of using the distant feeding spots was likely better able to differentiate risk‐taking individuals that show high and repeated encounters with baited hooks from risk‐averse individuals, in turn increasing predictive power.

### 4.1 Selection on behavior and life history

We showed that boldness in ponds is a dominant trait under selection in passive angling fisheries for carp. These results are in contrast to the findings of Monk and Arlinghaus (2017b) who did not find any correlation between repeatable large‐scale spatial or behavioral metrics such as activity space size, swimming distance, time spent within sublittoral, distance to the lake bottom, time at feeding sites, and switches between feeding sites and individual vulnerability to angling of common carp within a natural lake. By contrast, we found small‐scale spatial variation in risk‐taking behavior to be predictive for individual vulnerability of carp. Such behavior can be interpreted both as boldness (as the carp are able to sense the increasing risk of angling on feeding spots, Klefoth et al., 2012) and as a measure of bait encounters, which Monk and Arlinghaus (2017b) could not assess with the same degree of accuracy in their study. If these results hold for boldness in the wild, our work suggests that over time exploited populations of benthivorous fishes should be increasingly timid (hence the timidity syndrome, Arlinghaus et al., 2017) as observed in field studies of intensively exploited coastal fishes (Alós, Palmer, Trias, et al., 2015; Alós et al., 2016), largemouth bass within and outside protected areas (Twardek et al., 2017), and in a Japanese freshwater salmonid (Tsuboi et al., 2016). However, in our study a strong pattern of selection acting on our boldness measure only emerged in an angling period of 20 days and was not present immediately in the first week of angling. In fact, in our seven‐day fishing period, the selection pressures acting on total length (a surrogate for lifetime growth) were stronger than the strength of selection acting on boldness. As time progressed, increasing numbers of smaller, yet very bold individuals that visited the feedings spots were repeatedly hooked, “washing” down the selection pressure on length and growth rate and increasing the signal of selection acting on boldness.

Several factors may have contributed to variation in individual visits at the feeding spots (our boldness measure), such as variation in hunger (Thomson, Watts, Pottinger, & Sneddon, 2012; Vehanen, 2003), variation in threat perception (Brown, Jones, & Braithwaite, 2005), and variation in activity (Vehanen, 2003). These components of boldness may all have contributed to the predictive power of our boldness measure, but we were unable to precisely quantify them and disentangle the individual contributions. Independent of boldness selection, some selection continued to act on growth rate expressed in the ponds. It is very likely that fish with high growth rates not only visited the feeding spots more often but also consumed more particles once on a spot as previously documented for bold domesticated carp in comparison with shy wild‐like conspecifics (Klefoth et al., 2013). Results from piscivorous largemouth bass selected for their individual vulnerability confirm this assumption as highly vulnerable fish were shown to have higher prey capture success rates (Nannini, Wahl, Philipp, & Cooke, 2011). Growth rate also likely integrated the independent effects of unmeasured physiological and behavioral traits. For example, links among behavior, learning ability (DePasquale, Wagner, Archard, Ferguson, & Braithwaite, 2014; Kotrschal et al., 2014; Trompf & Brown, 2014), and metabolic rate (Biro & Stamps, 2010) have been reported in other studies (Hessenauer, Vokoun, Davis, Jacobs, & O′Donnell, 2016; Hessenauer et al., 2015), which may all affect growth rate (Redpath, Cooke, Arlinghaus, Wahl, & Philipp, 2009; Redpath et al., 2010). In line with Biro and Sampson (2015), we thus tentatively conclude that a sizable fraction of the remaining “direct” selection on juvenile growth rate can be explained by variation in unmeasured energy‐acquisition‐related behaviors (Enberg et al., 2012), for example, individual variation in intensity of ingesting baited hooks and freely available baits (Gutmann Roberts, Bašić, Amat Trigo, & Britton, 2017). Previous research in carp has indeed revealed that there is consistent individual variation in ingestion rates of seeds embedded in pellets (Pollux, 2017).

The negative selection gradients estimated on juvenile growth rate in the present study on first sight seem to support the “intuition” (Walters & Martell, 2004) that heavily exploited carp (and ecologically similar benthivorous species such as bream, *Abramis brama*, or tench, *Tinca tinca*) stocks should host individuals that grow less when adult, in line with empirical evidence in salmonids (Saura et al., 2010), esocids (Edeline et al., 2007), and several coastal and marine fishes (Alós, Palmer, Catalan, et al., 2014; Swain et al., 2007). However, our findings do not mean that evolution of reduced growth rate is a default response to intensive harvesting (see also Matsumura et al., 2011), because we found independent selection gradients acting on boldness and juvenile growth rate in carp and because we have no evidence of the direction and strength of natural selection pressures. Based on our work and a recent modeling study (Andersen et al., 2017), an evolutionary response to intensive harvesting of just boldness, just growth rate, or both is possible depending on the local fitness landscape and the degree to which natural selection works in opposite directions to fishing selection (Edeline et al., 2007). Indeed, the natural fitness benefits of fast growth and large size might easily overrule any angling‐induced negative selection gradients acting directly or indirectly on juvenile growth rate (Matsumura et al., 2011). For example, if there is a strong natural predation pressure on small‐bodied carp individuals, it is well possible that this creates large selection gradients toward large size that are greater than the negative selection gradients on growth rate documented here. If this is the case, the selection gradient on boldness should remain, and the evolution of timidity without a necessary change in growth is a possible outcome (Andersen et al., 2017; Arlinghaus et al., 2017). In fact, it is well possible that both fisheries and natural selection favors shyness in juvenile fishes (Ballew, Mittelbach, & Scribner, 2017). Only species‐ and fishery‐specific models that account for the lifetime fitness of specific trait values and the correlations among traits can provide conclusive answers (Laugen et al., 2014). Before this research becomes available, depending on the species, fisheries‐induced selection of either fast, slow, or no change in juvenile growth rate can all happen (Dunlop, Heino, & Dieckmann, 2009; Enberg et al., 2012; Matsumura et al., 2011), but evolution of timidity is most likely if boldness increases the likelihood of capture (Andersen et al., 2017; Arlinghaus et al., 2017). We would thus predict that the most consistent response to intensive harvesting in response to passive gear is the evolution of timidity (Andersen et al., 2017; Arlinghaus et al., 2017).

### 4.2 Selection on size

The lack of selection on size in a longer term over 20 days of angling, as observed in our study, should not be over‐interpreted because we purposely used fish of a very narrow size range to maximize behavioral variation and to control for the undisputed importance of size for vulnerability to angling (e.g., Lennox et al., 2017; Lewin et al., 2006). Larger fish under natural conditions generally show higher swimming speeds (Stamps, 2007), have larger gape sizes, are often dominant (Jenkins, 1969), often have larger home ranges (Nash, Welsh, Graham, & Bellwood, 2015), and are characterized by larger absolute consumptive demands compared to smaller fish (Clarke & Johnston, 1999; Mittelbach, Ballew, & Kjelvik, 2014), likely leading to intrinsically larger vulnerability to passive angling gear in large compared to small individuals (Tsuboi et al., 2016). Carp are no exception: Beukema and DeVos (1974) observed larger‐than‐average carp from two replicated ponds to be 20%–30% more likely to be captured by angling than their smaller‐than‐average conspecifics from the same water bodies. One would thus expect selection on size to be present under natural conditions. However, similar to our long‐term fishery, Monk and Arlinghaus (2017b) did not find evidence for size selectivity in carp angling under natural conditions when a large size range was present. It is therefore possible that the lack of size selection reported here for a 20 angling days fishery in fact holds for carp in general.

### 4.3 Selection on body shape

The body shape of the fish as determined by geometric morphometrics only added little to the suite of phenotypes under selection in our angling fishery. Whereas Alós, Palmer, and Linde‐Medina (2014) found comparatively strong evidence for angling‐induced selection on large mouth size and streamlined bodies in a coastal fish, we could only detect small, yet significant effects of body shape and head size and mouth on an individual's fitness in a passive hook‐and‐line fishery. Direct physical interactions of the mouth with the fishing gear and the mechanics of hooking can explain why individuals with a larger mouth are more likely to be captured (Alós, Palmer, and Linde‐Medina 2014) as an increasing gape size facilities ingestion of the hook (Alós, Cerdà, Deudero, & Grau, 2008). Indeed, Rapp et al. (2008) found evidence that smaller hooks capture more and larger carp in a natural fishery, indicating that the mouth size in relation to hook size affects the mechanics of hooking. Relatedly, we found some evidence that larger heads and mouths positively influenced vulnerability of the fish. In contrast to Alós, Palmer, and Linde‐Medina (2014), however, we found some evidence of deeply bodied fish to be more likely to be captured. Deep bodies are indicative of domestication selection in carp, and more domesticated carp are on average more vulnerable to angling than less domesticated conspecifics because the domesticated ones take more risks and feed more (Beukema, 1969; Huntingford, 2004; Klefoth et al., 2012; Klefoth et al., 2013). In addition, our results indicate the strongest selection to act on bold behavior, and selection on correlated morphological properties might appear stronger in the absence of direct measures of behavior as in the case of Alós, Palmer, and Linde‐Medina, (2014).

### 4.4 Limitations

Our studies are confined to the semi‐natural conditions in our ponds and thus can only be generalized to natural populations of carp or other ecologically similar benthivorous fishes with care. However, we believe our results are robust to the choice of the supply of carp, which happened to come from a commercial hatchery and might thus suffer from domestication effects. Several reasons play a role. First, the parental fish were held under near‐natural pond conditions for more than two generations, which has been reported to cause re‐adaptation of wild‐like behavior in common carp (Matsuzaki, Mabuchi, Takamura, Nishida, & Washitani, 2009). Second, the experimental carp were raised in a common‐garden environment where about 40 parental fish spawned naturally (i.e., no artificial mate choice or stripping), similar to what would happen in the wild. Based on the domestication history of parental fish, highly domesticated mirror carp and less domesticated scaled carp emerged from scaled parental fish, reflecting the genetics of scale pattern formation in carp (Kirpitchnikov, 1999). Third, previous research has revealed that the test fishes show very high behavioral diversity in semi‐natural ponds, with many individuals being entirely invulnerable to fishing, and domesticated and wild‐type common‐garden carp showing clear differences in boldness at the group level in the expected directions (Klefoth et al., 2012; Klefoth et al., 2013). Should the fish be highly domesticated, one would have expected that the vulnerability to fishing would have been excessive. But this was not the case with roughly half of the stock, particularly the wild‐type scaled carp, to be entirely invulnerable (Klefoth et al., 2012). Fourth, we tested behavioral scoring of personality in confined laboratory tanks and failed to relate behavior in tanks to the behavior in ponds and to angling vulnerability (Klefoth, 2017), confirming that the behavior expressed in the ponds represented nature‐like behavioral patterns. Despite all limitations, our study design has the strength that we used a representative subsample of nature‐like raised fish. Thus, we were able to avoid preselection based on trait selective capture techniques. We assume our test fish to represent some of the variation expected from natural populations of benthivorous fish.

## 5 CONCLUSIONS

In conclusion, our study is among the first in fishes to mechanistically show that selection on juvenile growth rate can happen as an indirect response to direct selection on behavior. Moreover, our work joins other recent findings (Alós et al., 2016) showing that behavioral traits might be under very strong selection in passively operated angling fisheries, but there is the caveat that a recent study by Monk and Arlinghaus (2017a) failed to document selection on feed patch use in the wild. We further found support for the productivity‐personality hypothesis (Biro & Stamps, 2008; Stamps, 2007), which predicts that boldness‐related behavior can be directly linked to resource acquisition and growth in omnivorous carp. The ultimate direction of the evolutionary response will depend on the heritability of the selected traits and on the relative strength of simultaneously acting natural and harvest selection (Edeline et al., 2007). Under natural conditions in repeat spawners, large body size often maximizes lifetime fitness (Alós, Palmer, Catalan, et al., 2014; Olsen & Moland, 2011; Roff, 1984), but there is an optimal growth rate to be expected given the unavoidable growth‐mortality trade‐off (Stamps, 2007). Because in omnivorous fishes like carp fast growth of early life stages should be favored to outgrow gape size limited predators and to maximize body size at first reproduction, the ultimate selection response of growth rate to positively size‐selective harvest will likely be weakened by natural selection working in the opposite direction (Edeline et al., 2007). However, we found boldness to be under strongest selection in our passive fishery and only a modest correlation of boldness and growth rate [in contrast to the crayfish data in Biro and Sampson (2015)]. Boldness may be less directly linked to lifetime reproductive fitness compared to size and growth, and indeed, the heritability of boldness and other behaviors has been found to be substantially greater compared to life history traits like growth (Dochtermann, Schwab, & Sih, 2015; Mousseau & Roff, 1987). Coupled with the strong selection gradients acting on boldness, we therefore predict that the evolutionary response of boldness‐related behaviors in response to recreational harvesting should be strong. As a consequence, intensive angling fisheries should leave behind individuals that are more timid and harder to catch (Philipp et al., 2009; Tsuboi et al., 2016), a pattern that might be further reinforced by learning to avoid future capture (Klefoth et al., 2013; Philipp et al., 2015), and by natural selection in juveniles also favoring shy fish (Ballew et al., 2017). This increased timidity (shyness) can have consequences for social groups, populations, and food webs and can negatively affect catchability and stock assessment (Alós, Palmer, Trias, et al., 2015; Alós, Puiggrós, et al., 2015; Arlinghaus et al., 2016, 2017; Tsuboi et al., 2016).

## DATA ARCHIVING STATEMENT

Data available from the Dryad Digital Repository: https://doi.org/10.5061/dryad.qj163
