# Supplementary material for: Toward a mechanistic understanding of vulnerability to hook‐and‐line fishing: Boldness as the basic target of angling‐induced selection
Source: Evol Appl. 2017 Aug 19;10(10):994–1006. doi: 10.1111/eva.12504 (PMC5680629; doi:10.1111/eva.12504)

Appendix 1

Morphological measurements of carp in our pond experiment.

Fig. 1: Body shape landmarks (N = 16) used for morphometric analysis in carp.

Fig. 2: Comparison of mean body shape of vulnerable (black line) and invulnerable (grey line) carp in a short-term (A, 7 d of angling) and long-term (B, 20 d of angling) passive angling fishery.

Fig. 1


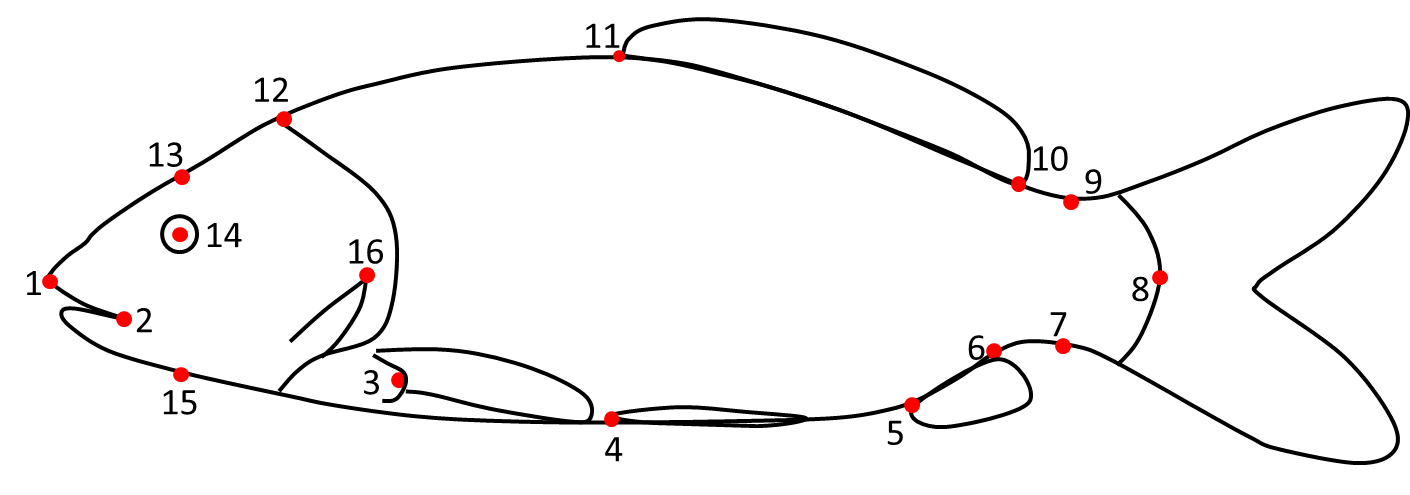


Fig. 2


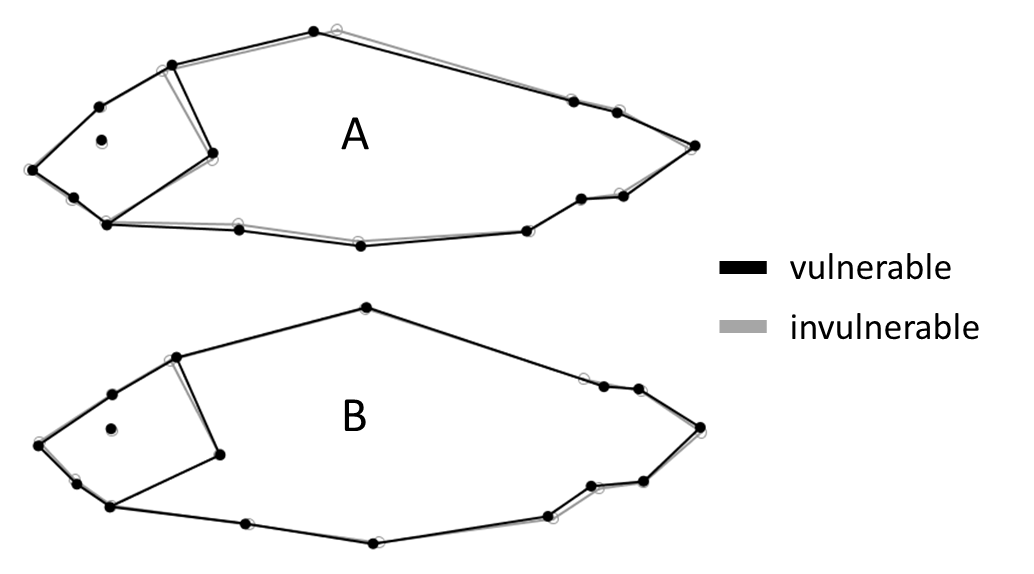


Appendix 2

Calibration of the PIT system

Calibration of the PIT system used in this study was conducted within a large laboratory tank. The size of the tank (10 m x 4 m x 1 m, L x W x H) and PIT system installation within the tank was similar to the setup used by Klefoth et al. (2012) and comparable to the setup within ponds used in the present study (compare Fig. 1 of the main manuscript). The PIT system covered two feeding spots (0.5 m diameter) and a shelter structure (2 m x 4 m). PIT antennae covering the feeding spots were round circle antennae where the antenna cable was bound to a circle and placed directly on the bottom of the tank. Two antenna loops (1 m x 4 m) were installed in front of the shelter structure and covered the complete width and height of the tank. We used N = 40 carp (TL ± SD = 228 ± 19.6 mm) tagged with PIT tags (23 mm, Oregon RFID, Oregon, USA) to calibrate our passive telemetry system. Out of the 40 fish, 3 randomly selected individuals were additionally tagged with external floy-tags (Floy Tag Inc., Seattle, Washington, USA) for external visual identification. The first individual was floy-tagged at the edge of the left pectoral fin (ID 348), the second individual was marked at the edge of both pectoral fins (ID 435), and the floy tag for the third individual was placed at the ventral point of maximum curvature of the peduncle (ID 351). These additional external tags allowed to identify three individuals to be present at the two feeding spots or to be present within the shelter structure, irrespective of the automatic PIT system and were therefore used for calibration of the PIT system.

Three cameras (Oscar CCD Camera 640 x 480 Pixel) were installed to visually observe the fish. Camera 1 and camera 2 were placed above the close and the distant feeding spot (approximately 0.5 m above the water surface) and camera 3 was installed at a window glass at the side of the tank where antennae loops in front of the shelter structure were located. On four consecutive days all N = 40 fish were simultaneously observed using the PIT system and the three cameras for one hour each. We followed the behavior of our three externally tagged individuals using the video data and compared the data with those collected from the automatic PIT system. Whenever an externally tagged carp entered one of the feeding spots (i.e. the fish was found to be within the antenna loop with a minimum of 50 % of its body length), we counted the maximum number of additional conspecifics within the feeding spot and measured the time spent within the antenna loop. Whenever one of the three focal fish reached one of the two antenna loops in front of the shelter structure we again counted the number of conspecifics present, but did not measure the time spent there as the single antenna loops in front of the shelter were only crossed during our observation periods and the focal fish did not stay on top of the antennae. We defined a fish to be sheltering after it had passed the antennae loops in a direction from outside to the inside of the shelter. Sheltering activities ended when the fish passed the antennae loops in the opposite direction or when it was detected elsewhere. Based on our video data we took eight randomly selected time frames of 10 minutes and stopped the time spent sheltering of our focal fish within this time frame. If a focal fish did not leave the shelter after the ten minutes, observation time was elongated until sheltering activities ended. We then compared theses real observations (min. spent sheltering) with our PIT data (min. spent sheltering). Similar comparisons were done for the feeding spots based on 18 randomly selected time frames of 10 minutes. Here, the number of visits at the feeding spots were counted on video data and compared with PIT data.

Statistics

To examine the functionality of the PIT system, logistic regression analyses were used. An individual observation was coded one if both, visual observations and the PIT system revealed a fish to be present within one of the structures (feeding spot, shelter structure). An observation was coded 0 if only the visual observation, but not the PIT system indicated a structure use of the fish. Visits at the close and the distant feeding spot were grouped as preliminary analyses revealed no differences in detection efficiency between these two similar structures. The number of conspecifics present at the same structure and the time (s) spent within the proximity of the antennae (only feeding spots) was added to the model as explanatory variable. The real time (min) spent sheltering and the real number of visits at the feeding spots as indicated by visual observations was compared with the time spent sheltering and the number of visits at feeding spots as calculated based on PIT data using Pearson`s correlation. Single events of specific habitat choice of the three externally marked fish were treated as independent observations. Analyses were conducted using software package R version 3.1.2 (R Development Core Team) by applying library MASS (Venables and Ripley 2002).

Results

During our one hour observation periods over four days we found the three focal fish to visit the feeding spots a total of 77 times. The mean time spent on the feeding spots was 3.1 ± 2.6 s (range 1 - 16 s) and the mean number of fish present was 3.2 ± 2.0 (range 1 – 10). The antennae in front of the shelter structure were crossed 395 times and the mean number of 5.0 ± 3.0 fish were present (range 0 – 15). The logistic regression analyses revealed the number of conspecifics present within the vicinity of the antennae to be the main predictor of PIT detection (Tab. 1, Tab. 2, Fig. 1, Fig. 2) whereas the time spent within the circle antennae loops did not influence detection probabilities (Tab. 2). Visual observations in comparison with our PIT data revealed a detection probability of 68.8 % over all cases at the circle antennae with a 100 % probability of detection if the focal fish was alone and continuously lower detection probabilities with an increasing number of conspecifics in the vicinity of the antennae (Tab. 1, Fig. 1). Our additional ten minutes observation periods of feeding spots (N = 18) revealed a high correlation between PIT system data and visual observations (Pearson`s correlation, T = 5.741, df = 16, r = 0.820, P < 0.001, Fig. 3). Similar observations of sheltering activities (N = 8) underestimated the time spent sheltering by factor 4.4 [mean time spent sheltering (min) ± SD based on visual observations and calculated from PIT data, 6.70 ± 2.84 and 1.52 ± 1.91 min, respectively], but were highly correlated with the real time spent sheltering as determined visually (Pearson`s correlation, T = 3.169, df = 6, r = 0.791, P = 0.019, Fig. 4). Therefore, the automatic PIT system revealed reliable data to observe the number of visits at feeding spots and the time spent sheltering within our experimental setup. However, the system was influenced by the number of conspecifics present and circle antennae on the bottom as used for covering the feeding spots revealed better results compared to antenna loops in front of the shelter structure.

References Appendix 2

Klefoth, T., C. Skov, J. Krause, and R. Arlinghaus, R. 2012. [The role of ecological context and predation risk-stimuli in revealing the true picture about the genetic basis of boldness evolution in fish](http://link.springer.com/article/10.1007/s00265-011-1303-2). Behavioral Ecology and Sociobiology 66:547-559.

Venables, W. N., and B. B. Ripley. 2002. *Modern applied statistics with S*. Springer, New York.

Tables Appendix 2

Tab. 1: Descriptive data of our calibration experiments. Presented are the total number of fish present when one of the focal fish used a PIT covered structure and the number of successful detections in dependence of conspecifics occurrence.

| Number of fish present | Number of observations | Positive detections | No detections | Proportion of positive detections % |
| --- | --- | --- | --- | --- |
| **@ Feeding spots** |  |  |  |  |
| 1 | 16 | 16 | 0 | 100 |
| 2 | 16 | 13 | 3 | 81 |
| 3 | 20 | 13 | 7 | 65 |
| 4 | 9 | 7 | 2 | 78 |
| 5 | 6 | 2 | 4 | 33 |
| 6 | 3 | 2 | 1 | 67 |
| 7 - 10 | 7 | 0 | 7 | 0 |
|  |  |  |  |  |
| **@ Shelter structure** |  |  |  |  |
| 1 | 33 | 28 | 5 | 85 |
| 2 | 50 | 23 | 27 | 46 |
| 3 | 60 | 7 | 53 | 12 |
| 4 | 50 | 1 | 49 | 2 |
| 5 | 59 | 4 | 55 | 7 |
| 6 | 43 | 1 | 42 | 2 |
| 7 - 16 | 100 | 0 | 100 | 0 |

Tab. 2: Logistic regression modelling of carp detection along two different types of structures (feeding spots and shelter) using an automatic PIT system within a laboratory tank. Independent parameters were the number of conspecifics present within the vicinity of the PIT antenna and the time (s) spent on feeding spots.

| Parameter | Estimate | SE | z-value | P |
| --- | --- | --- | --- | --- |
| **Feeding spots** |  |  |  |  |
| Conspecifics | -0.76 | 0.19 | -3.93 | < 0.001 |
| Time | 0.18 | 0.16 | 1.12 | 0.261 |
|  |  |  |  |  |
| **Shelter** |  |  |  |  |
| Conspecifics | -1.29 | 0.17 | -7.68 | < 0.001 |

Figures Appendix 2

Fig. 1: Probability of positive detection on feeding spots using an automatic PIT system and circle antennae placed at the bottom of a large tank in dependence of the number of conspecifics present.

Fig. 2: Probability of positive detection in front of a shelter structure using an automatic PIT system and antenna loops in dependence of the number of conspecifics present.

Fig. 3: Correlation between the real number of visits at the feeding spots during N = 18 ten minutes lasting periods as observed visually and the number of visits at the feeding spots as determined by PIT data.

Fig. 4: Correlation between the real time spent sheltering (min) during N = 8 ten minutes lasting periods as observed visually and the time spent under shelter (min) as determined by PIT data.

Fig. 1:


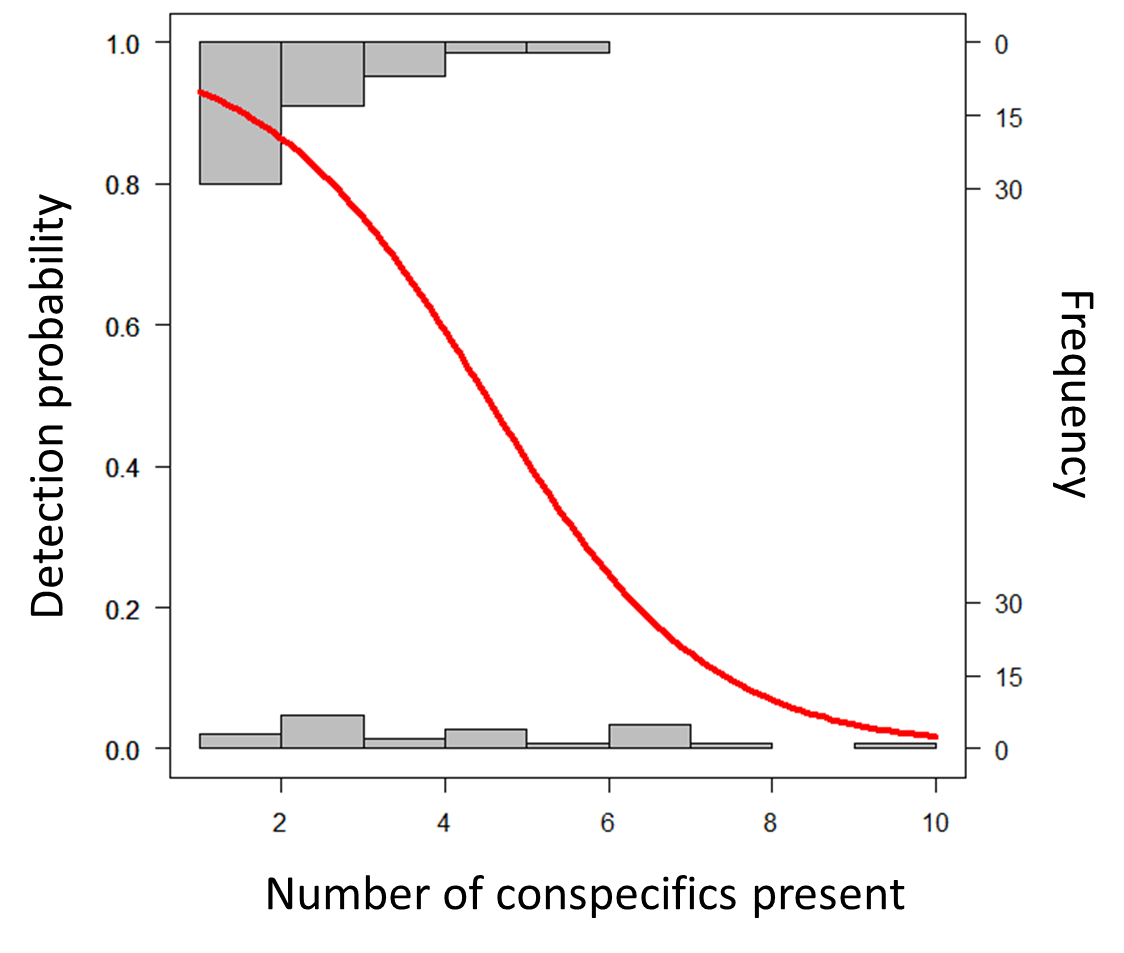


Fig. 2:


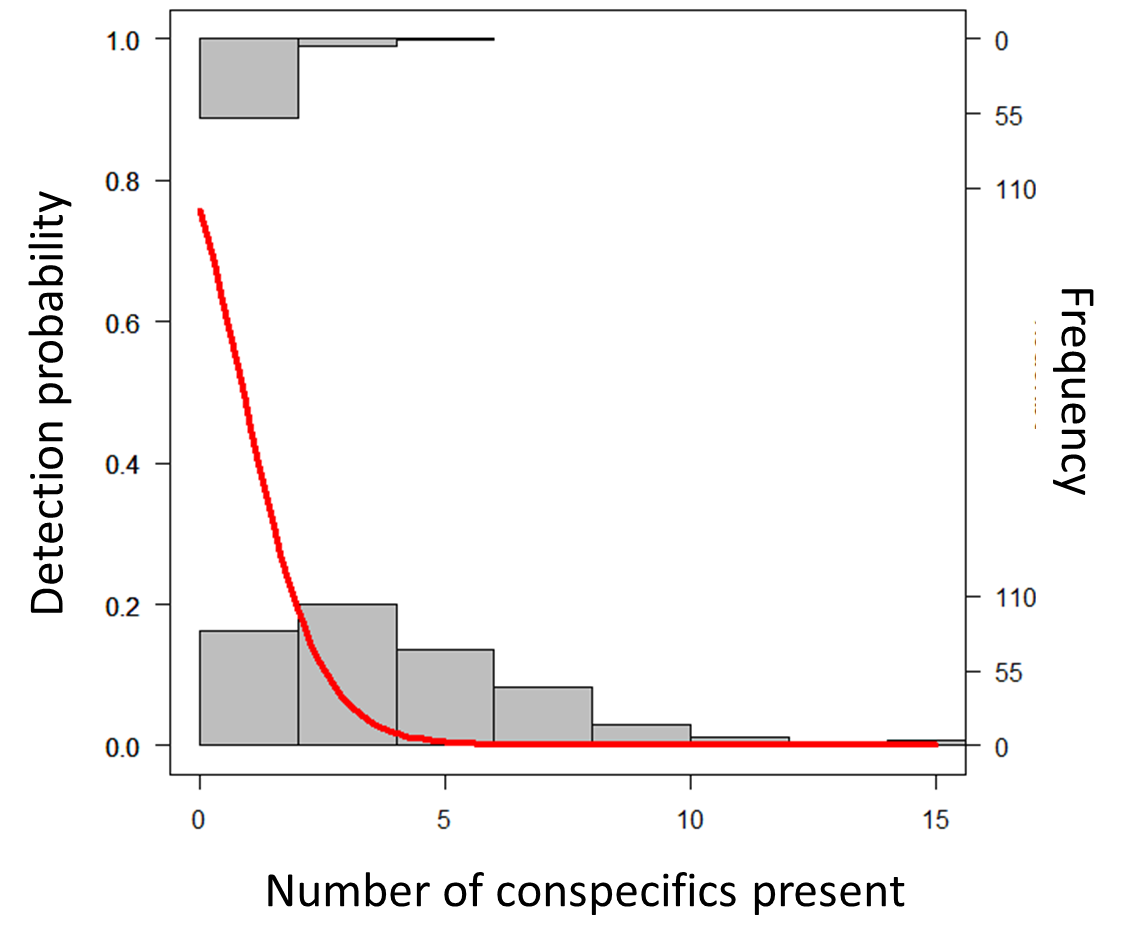


Fig. 3:


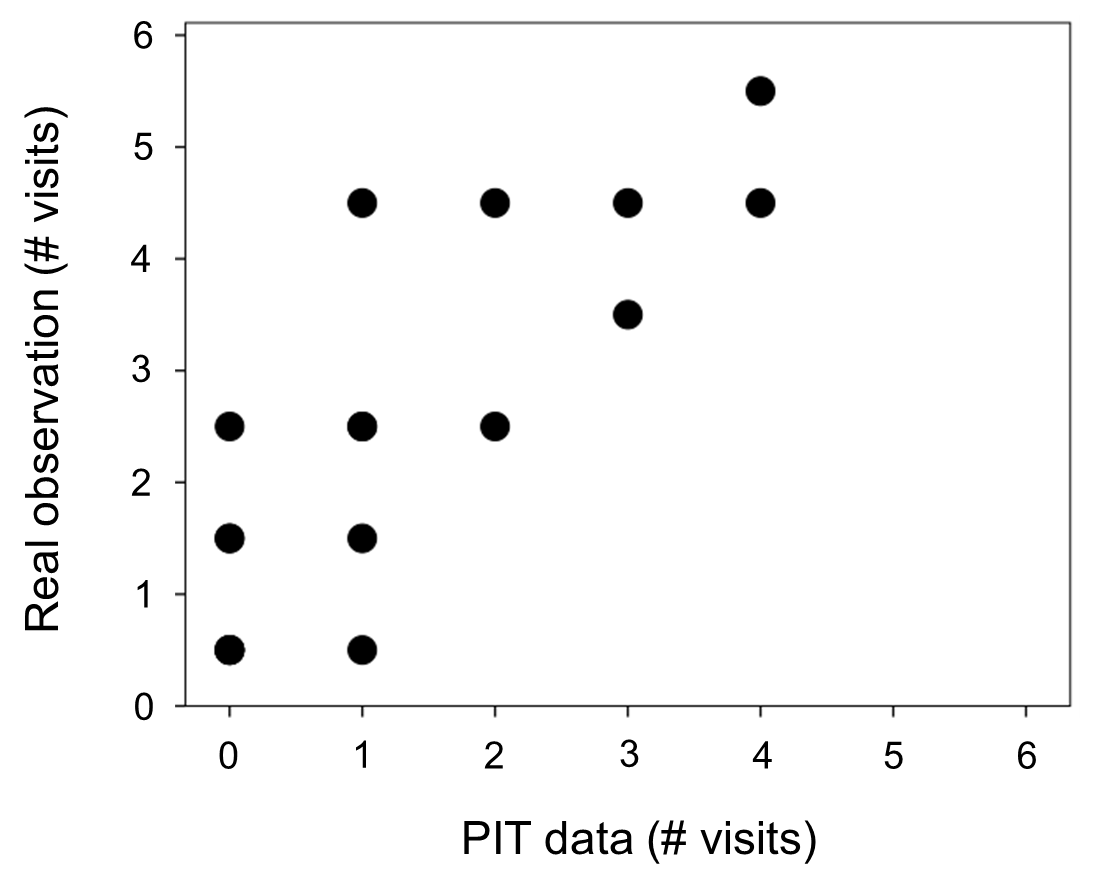


Fig. 4:


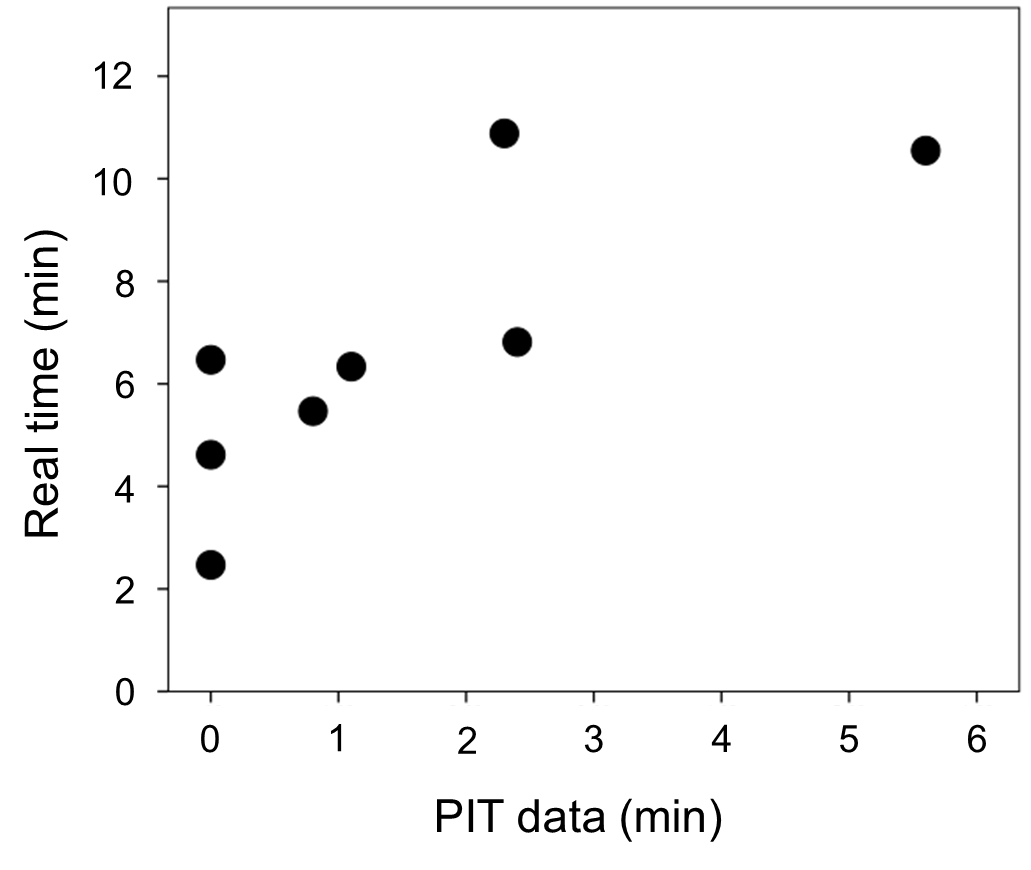

Supplement: Supplementary file 1 [file EVA-10-994-s001.docx]
